# Serum Hemoglobin-to-Creatinine Ratio and Post-Discharge Readmission or Mortality in Older Patients With Heart Failure: A Retrospective Cohort Study

**DOI:** 10.31083/RCM45668

**Published:** 2026-05-06

**Authors:** Hong-Bo Xu, Ying He, Yu-Hong Xu, Hai-Gang Zhang

**Affiliations:** ^1^Department of Critical Care Medicine, Shenzhen Nanshan People's Hospital, and Affiliated Nanshan Hospital of Shenzhen University, 518052 Shenzhen, Guangdong, China; ^2^Department of Laboratory Medicine, The Eighth Affiliated Hospital, Sun Yat-Sen University, 518033 Shenzhen, Guangdong, China; ^3^Department of Pharmacy, The Eighth Affiliated Hospital, Sun Yat-Sen University, 518033 Shenzhen, Guangdong, China

**Keywords:** heart failure, anemia, renal insufficiency, prognosis

## Abstract

**Background:**

Older patients with heart failure (HF) experience poor prognoses after hospital discharge, underscoring the importance of risk stratification for improving out-of-hospital management. Therefore, this study aimed to investigate the association between a composite index of anemia and renal impairment, defined as the serum hemoglobin-to-creatinine ratio (HCR), and post-discharge readmission or mortality in older patients with HF.

**Methods:**

Data were obtained from the Zigong Fourth People's Hospital. HF was diagnosed in accordance with the 2016 European Society of Cardiology guidelines. The HCR was employed, while the outcome was a composite of readmission or mortality assessed at 180 and 90 days after discharge. The association between the HCR and outcomes was analyzed using Cox proportional hazards models, with robustness evaluated through subgroup and sensitivity analyses.

**Results:**

The study cohort included 1781 older patients (age ≥60 years) with HF, of whom 41.6% experienced readmission or mortality within 180 days after discharge. Multivariable Cox regression indicated that a higher HCR was associated with a lower risk of 180-day readmission or mortality (hazard ratio (HR) = 0.76; 95% confidence interval (CI): 0.67–0.87). When analyzed using the HCR tertiles, the middle and highest tertiles exhibited progressively reduced risks (HR = 0.74, 95% CI: 0.61–0.88 and HR = 0.58, 95% CI: 0.47–0.71), respectively; *p* for trend < 0.001) compared with the lowest tertile. Similar associations were observed for the 90-day composite outcome. The stability of these associations was confirmed through subgroup and sensitivity analyses.

**Conclusions:**

A higher HCR is independently associated with a reduced risk of readmission or mortality within 180 days of discharge in older patients with HF. The HCR may serve as a useful prognostic marker for risk stratification in this population.

## 1. Introduction

Heart failure (HF) is a global health issue, affecting over 64 million people worldwide. It is a major cause of morbidity and mortality, resulting in significant healthcare expenditures that place considerable strain on individuals and society as a whole [1]. The condition primarily impacts the elderly, with incidence and prevalence rising sharply among adults over 60 years of age [2]. Although advances in medical care have reduced in-hospital mortality, HF patients continue to experience high rates of post-discharge readmission and mortality [3, 4]. Therefore, identifying high-risk patients after discharge is essential for optimizing management strategies and improving long-term outcomes.

Anemia and renal dysfunction are prevalent comorbidities in HF, and both have been linked to adverse outcomes, including increased mortality, hospital readmission, and prolonged hospitalization [5, 6, 7, 8]. It is important to note that these two conditions frequently coexist and interact. Renal impairment can directly contribute to anemia, for example, in cases of renal anemia, while low hemoglobin levels may in turn exacerbate renal dysfunction [9, 10]. It has been suggested that anemia and renal impairment may synergistically accelerate HF progression, leading to a worsened prognosis [11]. We therefore hypothesize that a composite indicator of anemia and impaired renal function is associated with adverse outcomes in HF patients. Serum hemoglobin and creatinine levels are readily available laboratory parameters commonly used to assess anemia and renal function, and are routinely evaluated in HF patients [12]. Therefore, this study aimed to assess the association between the composite indicator, serum hemoglobin-to-creatinine ratio (HCR), and post-discharge readmission or mortality in a cohort of elderly patients with HF.

## 2. Methods

### 2.1 Data Source

This retrospective cohort study utilized an open-access database of hospitalized HF patients, which integrated electronic medical records with follow-up information [13]. The database comprises anonymized records of adult patients admitted for HF at the Zigong Fourth People’s Hospital (Sichuan, China), between December 2016 and June 2019. Available data include demographics, comorbidities, vital signs, laboratory parameters, in-hospital treatments, and post-discharge outcome. HF diagnoses in this database adhered to the 2016 European Society of Cardiology guidelines [13, 14]. After completing the online training course, author HBX (record ID 35959043) obtained approval to access the database for research. The study was conducted in accordance with the Strengthening the Reporting of Observational Studies in Epidemiology (STROBE) guidelines.

### 2.2 Study Population

**Fig. 1.**
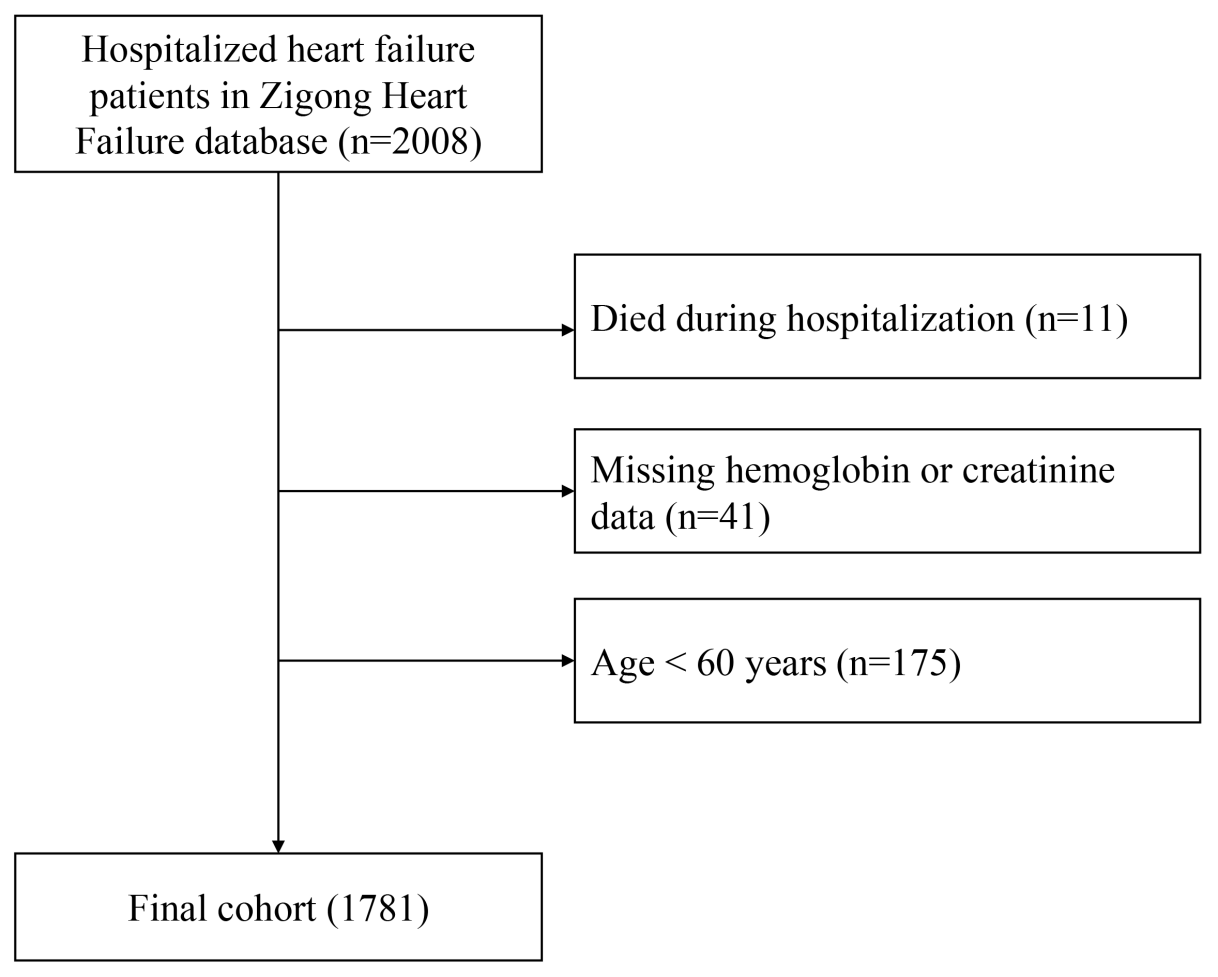
**Flow chart of study population selection**.

The initial cohort comprised all patients (n = 2008) from the database. We excluded individuals who met any of the following criteria: death during hospitalization, missing hemoglobin or creatinine values, or age below 60 years. Fig. 1 presents the patient selection flowchart.

### 2.3 Variables

Data extracted from the database encompassed demographic characteristics, clinical characteristics, comorbidities, laboratory parameters, and medication records. Demographic information included age, sex, and body mass index (BMI). Baseline clinical characteristics consisted of heart rate, respiratory rate, temperature, mean arterial pressure (MAP), New York Heart Association (NYHA) classification, left ventricular ejection fraction (LVEF), and Charlson Comorbidity Index (CCI). Documented comorbidities included diabetes, chronic kidney disease (CKD), chronic obstructive pulmonary disease (COPD), dementia, and cerebrovascular disease. Laboratory results at admission covered sodium, potassium, white blood cell count, hemoglobin, platelet count, albumin, bilirubin, creatinine, high-sensitivity troponin (hs-troponin), and brain natriuretic peptide (BNP). Medication use during hospitalization recorded loop diuretics, renin-angiotensin-aldosterone system (RAAS) inhibitors, spironolactone, beta-blockers, inotropes, statins, and antiplatelet agents.

### 2.4 Exposure and Outcome

The HCR exposure factor was calculated as hemoglobin (g/L) divided by creatinine (µmol/L) [14]. The primary endpoint was the composite outcome of all-cause readmission or mortality within 180 days after hospital discharge. The secondary outcome was defined as the same composite endpoint assessed at 90 days post-discharge. Outcome data were obtained through mandatory follow-up visits or telephone as specified in the database protocol.

### 2.5 Management of Missing Values

Several variables in this study contained missing values: including albumin, bilirubin, hs-troponin, BNP, sodium, potassium, CCI, and LVEF. Among these only LVEF had a high proportion of missing data at 68.73%, while the remainder exhibited missing rates below 5%. Due to the established importance of LVEF in HF prognosis, we incorporated it as a dummy variable with a category designating the missing status. For the other variables, missing values were imputed using the median or mean, as appropriate.

### 2.6 Statistical Analysis

The study population was categorized into three groups based on HCR for descriptive analysis. Continuous variables are expressed as mean ± standard deviation (SD) or median (interquartile range [IQR]) and compared by one-way analysis of variance (ANOVA) test or Kruskal-Wallis test, as appropriate. Categorical variables are presented as numbers (percentages) and analyzed using the Chi-square or Fisher’s exact test. Kaplan–Meier curves depicted composite outcomes across the three groups, and the log-rank test compared cumulative event incidence. The association between HCR and outcomes was evaluated through unadjusted and multivariable-adjusted Cox proportional hazards models, with results expressed as hazard ratios (HR) and 95% confidence intervals (CI). The proportional hazards assumption was verified before Cox regression. Adjustment variables were selected based on clinical relevance, a univariate *p*-value below 0.1, and a change in effect estimate exceeding 10% [15]. Multicollinearity was evaluated using the variance inflation factor (VIF), with VIF >5 indicating its presence. Model 1 was unadjusted. Model 2 adjusted for age, sex and BMI. Model 3 further adjusted for CCI, LVEF, NYHA classification, MAP, diabetes, CKD, sodium, potassium, albumin, BNP, bilirubin, loop diuretics, RAAS inhibitors, beta-blockers, and inotropes. To detect the likelihood of non-linearity, HCR was converted into tertile-based categories and a trend *p*-value was computed. The relationship between HCR and post-discharge outcomes was also explored using restricted cubic splines. Subgroup analyses were conducted to assess the association’s robustness, including sex, age (<80, ≥80), BMI (<18.5, 18.5–24, ≥24), NYHA classification, CCI (<2, ≥2), diabetes, COPD, dementia, LVEF value (≥50%, <50%, and missing), and cerebrovascular disease. Three sensitivity analyses tested the robustness of primary findings: a complete-case analysis excluding individuals with missing data; exclusion of CKD patients; and restriction to patients with available LVEF data. Finally, Receiver operating characteristic (ROC) curve analysis was performed to assess the predictive value of HCR and BNP for 180-day mortality or readmission after hospital discharge, with the optimal cutoff value determined based on the Youden index. Data were analyzed using R software (version 4.2.1, R Foundation for Statistical Computing, Vienna, Austria) and Free Statistics software (version 1.7.1, Free Clinical Medical Technology Co., Ltd, Beijing, China). A two tailed *p*-value of 0.05 or less was deemed to be statistically significant.

## 3. Results

### 3.1 Baseline Characteristics

A total of 1781 patients met the selection criteria and were included in the final analysis. The baseline characteristics of the study population are summarized in Table 1. Overall, 41.0% of the population was over 80 years of age, and 40.4% of male. Within 90 days of hospital discharge, 27.0% of patients experienced readmission or mortality, a figure that increased to 41.6% by the 180-day follow-up. Compared to patients in the higher HCR tertiles (T2 and T3), those in the lowest HCR tertile (T1) were relatively older and more likely to be male. They also exhibited lower MAP, lower heart rate, poor cardiac function and higher CCI scores. Patients with lower HCR were more likely to have a history of CKD, along with higher creatinine levels and lower hemoglobin levels. Regarding other laboratory parameters, lower HCR correlated with higher potassium, lower sodium, lower albumin, lower bilirubin and higher BNP levels. Differences among the groups were also observed in the use of loop diuretics, RAAS inhibitors, spironolactone and beta-blockers.

**Table 1.** **Characteristics of the study population according to the tertiles of hemoglobin-to-creatinine ratio**.

Variables		Total	T1 (<1.03)	T2 (≥1.03, <1.65)	T3 (≥1.65)	*p*
		(n = 1781)	(n = 599)	(n = 594)	(n = 588)	
Age ≥80 y, n (%)		730 (41.0)	287 (47.9)	256 (43.1)	187 (31.8)	<0.001
Male, n (%)		720 (40.4)	291 (48.6)	267 (44.9)	162 (27.6)	<0.001
MAP, mmHg		95.0 ± 16.1	93.1 ± 16.5	95.0 ± 16.3	96.9 ± 15.3	<0.001
Heart rate, bpm		84.6 ± 21.5	80.7 ± 20.6	87.3 ± 22.3	85.8 ± 21.2	<0.001
Respiratory rate, bpm		19.1 ± 1.7	19.0 ± 1.7	19.1 ± 1.7	19.0 ± 1.7	0.563
Temperature, °C		36.4 ± 0.4	36.4 ± 0.5	36.4 ± 0.4	36.4 ± 0.4	0.207
BMI (kg/m^2^)		20.8 (18.4, 23.4)	20.6 (18.4, 23.4)	20.7 (18.4, 23.1)	21.1 (18.5, 23.5)	0.151
NYHA classification, n (%)						<0.001
	II	309 (17.3)	84 (14.0)	106 (17.8)	119 (20.2)	
	III	918 (51.5)	290 (48.4)	300 (50.5)	328 (55.8)	
	IV	554 (31.1)	225 (37.6)	188 (31.6)	141 (24.0)	
LVEF, n (%)						0.003
	≥50%	312 (17.5)	91 (15.2)	102 (17.2)	119 (20.2)	
	<50%	245 (13.8)	68 (11.4)	78 (13.1)	99 (16.8)	
	Missing	1224 (68.7)	440 (73.5)	414 (69.7)	370 (62.9)	
CCI, n (%)						<0.001
	<2	710 (39.9)	175 (29.2)	250 (42.1)	285 (48.5)	
	≥2	1071 (60.1)	424 (70.8)	344 (57.9)	303 (51.5)	
Comorbidities, n (%)						
	Diabetes	422 (23.7)	160 (26.7)	128 (21.5)	134 (22.8)	0.091
	CKD	434 (24.4)	298 (49.7)	111 (18.7)	25 (4.3)	<0.001
	COPD	220 (12.4)	66 (11.0)	83 (14.0)	71 (12.1)	0.291
	Dementia	112 (6.3)	31 (5.2)	38 (6.4)	43 (7.3)	0.314
	Cerebrovascular disease	131 (7.4)	39 (6.5)	47 (7.9)	45 (7.7)	0.614
Laboratory tests						
	Sodium (mmol/L)	138.4 ± 4.9	137.1 ± 5.0	138.7 ± 4.7	139.2 ± 4.8	<0.001
	Potassium (mmol/L)	4.0 ± 0.7	4.3 ± 0.8	3.9 ± 0.6	3.8 ± 0.6	<0.001
	White blood cell (10^9^/L)	6.4 (5.0, 8.6)	6.2 (4.8, 8.5)	6.5 (5.1, 8.8)	6.4 (5.2, 8.2)	0.124
	Hemoglobin (g/L)	113.7 ± 24.0	96.5 ± 24.1	118.8 ± 19.5	126.1 ± 16.8	<0.001
	Platelet (10^9^/L)	133.0 (99.0, 172.0)	131.0 (95.5, 178.0)	134.0 (96.0, 170.0)	134.0 (104.0, 170.2)	0.721
	Albumin (g/L)	36.4 ± 4.7	35.0 ± 4.8	36.8 ± 4.5	37.6 ± 4.5	<0.001
	Bilirubin (µmol/L)	17.9 (12.2, 26.7)	15.5 (10.1, 25.3)	18.9 (13.4, 27.7)	18.1 (13.5, 26.6)	<0.001
	Creatinine (µmol/L)	88.7 (65.7, 124.2)	146.5 (120.8, 199.8)	90.4 (79.1, 102.1)	60.8 (52.3, 68.7)	<0.001
	BNP (pg/mL)	766.2 (319.4, 1722.0)	1001.0 (412.9, 2235.0)	778.8 (393.5, 1775.0)	524.4 (212.4, 1231.0)	<0.001
	hs-troponin (pg/mL)	0.1 (0.0, 0.1)	0.1 (0.0, 0.2)	0.1 (0.0, 0.1)	0.0 (0.0, 0.1)	<0.001
Medication, n (%)						
	Loop diuretics	1719 (96.5)	587 (98.0)	579 (97.5)	553 (94.0)	<0.001
	RAAS inhibitor	665 (37.3)	174 (29.0)	252 (42.4)	239 (40.6)	<0.001
	Spironolactone	1624 (91.2)	520 (86.8)	560 (94.3)	544 (92.5)	<0.001
	Beta-blocker	658 (36.9)	179 (29.9)	238 (40.1)	241 (41.0)	<0.001
	Inotropes	1293 (72.6)	429 (71.6)	445 (74.9)	419 (71.3)	0.298
	Statin	768 (43.1)	267 (44.6)	258 (43.4)	243 (41.3)	0.519
	Antiplatelet drugs	1114 (62.5)	385 (64.3)	378 (63.6)	351 (59.7)	0.212

Data are shown as the mean (standard deviation), median (interquartile range), or numbers (%). Abbreviations: MAP, mean arterial pressure; BMI, body mass index; NYHA classification, New York Heart Association classification; LVEF, left ventricular ejection fraction; CCI, Charlson Comorbidity Index; CKD, chronic kidney disease; COPD, chronic obstructive pulmonary disease; BNP, brain natriuretic peptide; hs-troponin, high sensitivity troponin; RAAS, renin-angiotensin-aldosterone system.

### 3.2 Association Between HCR and Post-Discharge Readmission or Mortality 

**Fig. 2.**
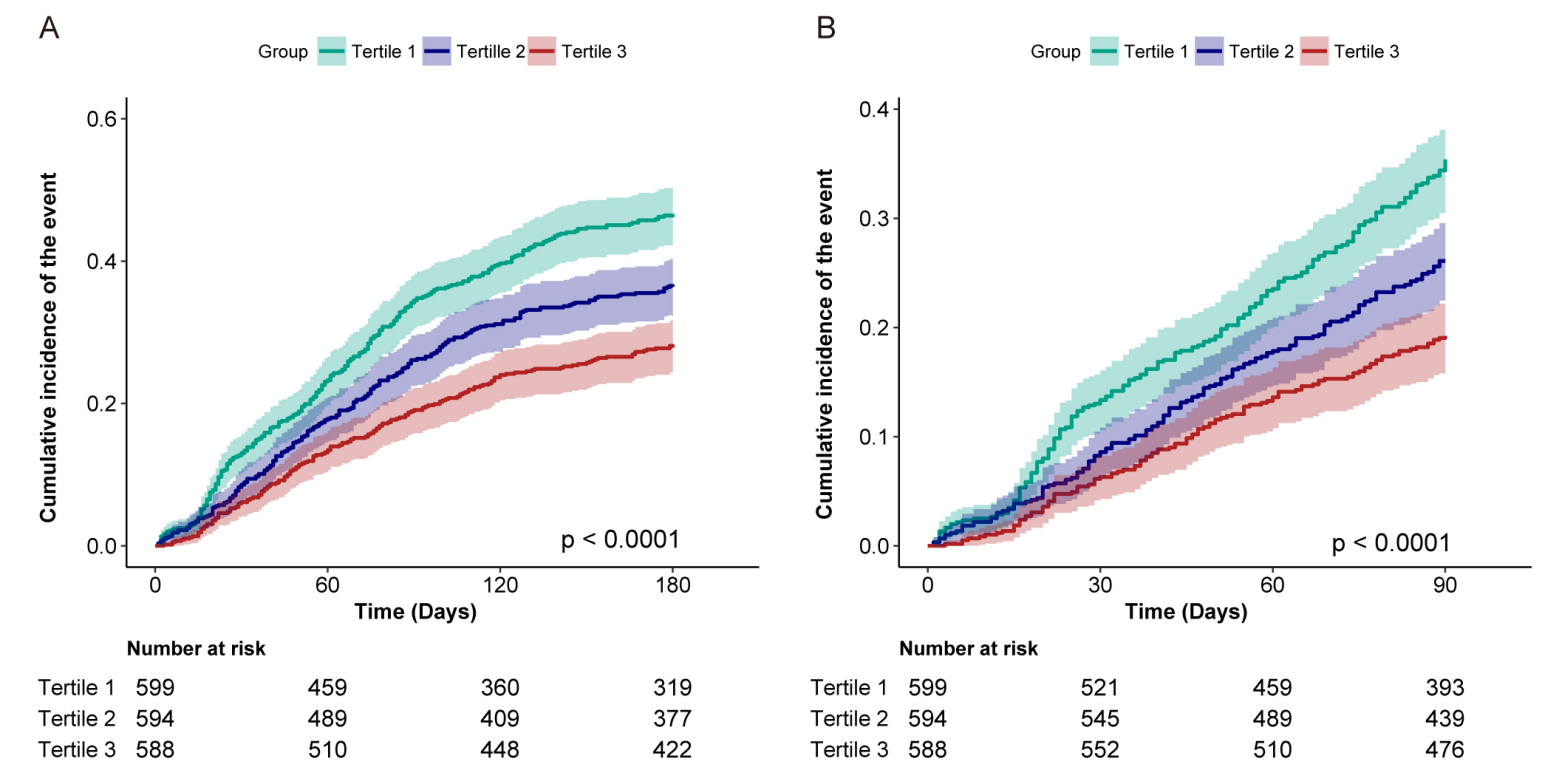
**Cumulative incidence of post-discharge readmission or mortality according to the tertile of HCR**. (A) Readmission or mortality within 180 days after hospital discharge. (B) Readmission or mortality within 90 days after hospital discharge. HCR, hemoglobin-to-creatinine ratio.

The cumulative incidence of readmission or mortality within 180 days after discharge was evaluated across HCR tertiles. Patients with lower HCR exhibited a higher rate of readmission or mortality (log-rank *p* < 0.0001; Fig. 2A). A similar pattern was observed for the 90-day post-discharge outcome (log-rank *p* < 0.0001; Fig. 2B).

Unadjusted and adjusted Cox regression models were employed to further evaluate the association between HCR and post-discharge outcomes (Table 2). In univariable analysis, higher HCR was significantly associated with a lower risk of 180-day composite outcome (Model 1: HR = 0.72, 95% CI: 0.65–0.81, *p* < 0.001). The inverse association persisted in multivariable models (Model 2: HR = 0.72, 95% CI: 0.64–0.81, *p* < 0.001; Model 3: HR = 0.76, 95% CI: 0.67–0.87, *p* < 0.001). When analyzed categorically by tertile, both tertile 2 (HR = 0.74, 95% CI: 0.61–0.88, *p* = 0.001) and tertile 3 (HR = 0.58, 95% CI: 0.47–0.71, *p* < 0.001) showed a lower risk of the composite endpoint compared with tertile 1, after adjusting for age, sex, BMI, CCI, LVEF, NYHA classification, MAP, diabetes, CKD, sodium, potassium, albumin, BNP, bilirubin, loop diuretics, RAAS inhibitor, beta blocker, and inotropes. Linear trend tests across tertiles were statistically significant in all three models. A comparable relationship was observed between HCR and 90-day composite outcome.

**Table 2.** **Results of Cox regression between HCR and composite outcome in patients with heart failure**.

		Event (%)	Model 1		Model 2		Model 3	
			HR (95% CI)	*p* value	HR (95% CI)	*p* value	HR (95% CI)	*p* value
180-day readmission or mortality after hospital discharge								
	HCR	741 (41.6)	0.72 (0.65–0.81)	<0.001	0.72 (0.64–0.81)	<0.001	0.76 (0.67–0.87)	<0.001
Tertiles of HCR								
	T1	307 (51.3)	Reference		Reference		Reference	
	T2	242 (40.7)	0.72 (0.61–0.86)	0.001	0.72 (0.61–0.85)	<0.001	0.74 (0.61–0.88)	0.001
	T3	192 (32.7)	0.54 (0.45–0.65)	<0.001	0.54 (0.45–0.65)	<0.001	0.58 (0.47–0.71)	<0.001
	*p* for trend			<0.001		<0.001		<0.001
90-day readmission or mortality after hospital discharge								
	HCR	480 (27.0)	0.71 (0.61–0.81)	<0.001	0.71 (0.62–0.82)	<0.001	0.85 (0.72–1.00)	0.052
Tertiles of HCR								
	T1	212 (35.4)	Reference		Reference		Reference	
	T2	155 (26.1)	0.69 (0.56–0.85)	0.001	0.69 (0.56–0.85)	<0.001	0.74 (0.59–0.92)	0.007
	T3	113 (19.2)	0.49 (0.39–0.62)	<0.001	0.49 (0.39–0.62)	<0.001	0.58 (0.45–0.75)	<0.001
	*p* for trend			<0.001		<0.001		<0.001

Model 1 adjusted for nothing; Model 2 adjusted for age, sex, and BMI; Model 3 adjusted for age, sex, BMI, CCI, LVEF, NYHA classification, MAP, diabetes, CKD, sodium, potassium, albumin, BNP, bilirubin, loop diuretics, RAAS inhibitor, beta blocker, inotropes. Abbreviations: HCR, hemoglobin-to-creatinine ratio; HR, Hazard ratio; BMI, body mass index; CI, confidence interval; CCI, Charlson Comorbidity Index; LVEF, left ventricular ejection fraction; NYHA, New York Heart Association; MAP, mean arterial pressure; CKD, chronic kidney disease; BNP, brain natriuretic peptide; RAAS, renin-angiotensin-aldosterone system.

**Fig. 3.**
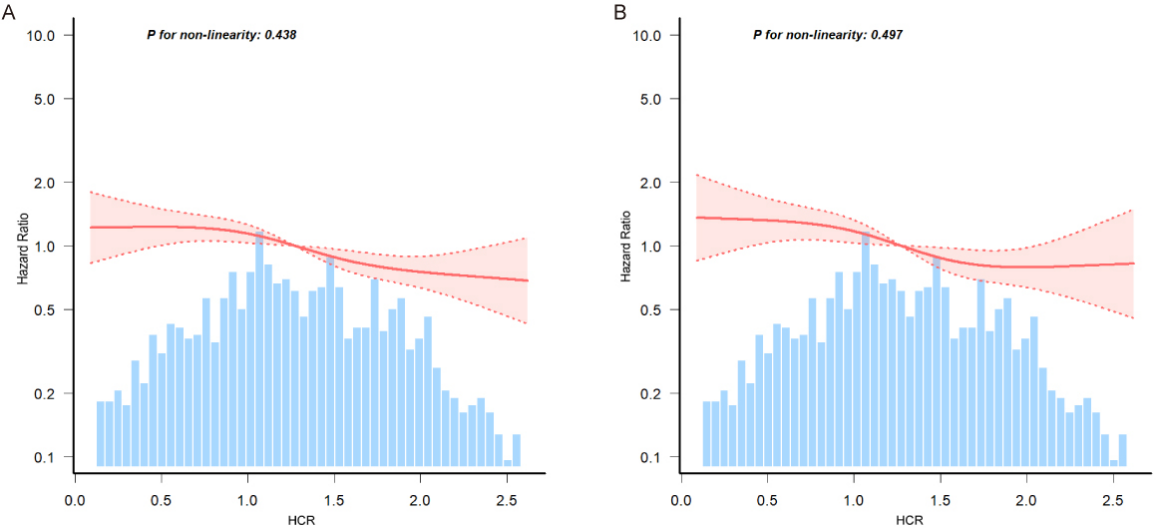
**Association between HCR and 180-day (A) and 90-day (B) post-discharge composite outcomes (mortality or readmission) in elderly HF patients**. Data were fitted by a multivariable-adjusted restricted cubic spline Cox’s regression. A linear association between HCR and 180-day and 90-day post-discharge composite outcomes was observed. HCR was entered as a continuous variable, the variables in model 3 of Table 2 were adjusted. The curves line and shaded ribbons around represented the estimated values and their corresponding 95% confidence intervals. HF, heart failure.

Multivariable-adjusted restricted cubic spline analysis revealed a linear association between HCR and 180-day as well as 90-day readmission or mortality in elderly heart failure patients (Fig. 3). Given the linearity of the associations, the effect sizes are appropriately summarized as hazard ratios per 1-unit increase in HCR. In the fully adjusted model (Model 3), each 1-unit increase in HCR was associated with a 24% reduction in the risk of 180-day mortality or readmission after discharge (HR = 0.76, 95% CI: 0.67–0.87) and a 15% reduction in the risk of the 90-day composite outcome (HR = 0.85, 95% CI: 0.72–1.00).

### 3.3 Subgroup and Sensitivity Analyses 

Stratified analyses of the association between HCR and 180-day post-discharge outcomes are presented in Table 3. A significant interaction was observed for age (*p* for interaction < 0.05). The respective HRs for the 60–80 years and over-80 years groups were 0.67 (95% CI: 0.57–0.81) and 1.13 (95% CI: 0.90–1.43). No other significant interactions were detected across the remaining subgroups, including sex, BMI (<18.5, 18.5–24, ≥24), NYHA classification, CCI (<2, ≥2), diabetes, COPD, dementia, LVEF value (≥50%, <50%, and missing), and cerebrovascular disease (all *p* for interaction > 0.05). Similar results were observed in subgroup analyses for the secondary endpoint of 90-day readmission or mortality after discharge (**Supplementary Table 1**).

**Table 3.** **Subgroup analysis of the association of HCR and 180-day readmission or mortality after hospital discharge**.

Subgroup		Events (%)	HR (95% CI)	*p* for interaction
Sex				
	Female	427 (40.2)	0.81 (0.68–0.95)	0.054
	Male	314 (43.6)	0.75 (0.57–0.97)	
Age				
	≥80	299 (41.0)	1.13 (0.90–1.43)	0.006
	<80	442 (42.1)	0.67 (0.57–0.81)	
BMI				
	<18.5	199 (43.0)	0.83 (0.63–1.09)	0.936
	18.5–24	391 (41.2)	0.81 (0.66–0.99)	
	≥24	151 (41.0)	0.81 (0.60–1.09)	
NYHA classification				
	II	101 (32.7)	0.71 (0.48–1.05)	0.197
	III	366 (39.9)	0.81 (0.67–0.97)	
	IV	274 (49.5)	0.92 (0.71–1.19)	
LVEF				0.574
	≥50%	129 (41.3)	0.71 (0.49–1.04)	
	<50%	105 (42.9)	0.81 (0.52–1.27)	
	Missing	507 (41.4)	0.82 (0.70–0.96)	
CCI				
	<2	281 (39.6)	0.69 (0.55–0.85)	0.254
	≥2	460 (43.0)	0.89 (0.74–1.06)	
Diabetes				
	No	539 (39.7)	0.73 (0.61–0.86)	0.221
	Yes	202 (47.9)	0.98 (0.76–1.25)	
COPD				
	No	653 (41.8)	0.82 (0.71–0.95)	0.347
	Yes	88 (40.0)	0.83 (0.51–1.34)	
Dementia				
	No	685 (41.0)	0.81 (0.70–0.94)	0.819
	Yes	56 (50.0)	0.66 (0.35–1.23)	
Cerebrovascular disease				
	No	686 (41.6)	0.77 (0.66–0.89)	0.444
	Yes	55 (42.0)	1.29 (0.74–2.22)	

Abbreviations: HCR, hemoglobin-to-creatinine ratio; HR, hazard ratio; CI, confidence interval; BMI, body mass index; NYHA classification, New York heart association classification; CCI, Charlson Comorbidity Index; COPD, chronic obstructive pulmonary disease; LVEF, left ventricular ejection fraction.

Further sensitivity analyses, which excluded patients with missing values, pre-existing CKD or missing LVEF values, also confirmed the stability of the association between HCR and readmission or mortality after hospital discharge (**Supplementary Tables 2,3,4**).

### 3.4 ROC Analysis

The ROC analysis results are included in the **Supplementary Materials** (**Supplementary Table 5, Supplementary Fig. 1**). The areas under the ROC curve (95% CI) for HCR and BNP were 0.585 (0.559–0.612) and 0.544 (0.516–0.571), respectively, with optimal cutoff values of 1.10 and 741.34. Additionally, the area under the ROC curve (95% CI) for the combination of HCR and BNP was 0.591 (0.565–0.618).

## 4. Discussion

This study investigated the association between HCR and post-discharge readmission or mortality in elderly HF patients in China, revealing that higher HCR correlated with a lower risk of the outcomes within 180 days after discharge. Subgroup and sensitivity analyses further confirmed the robustness of this association.

Anemia, characterized by reduced hemoglobin levels, is highly prevalent among HF patients, affecting approximately 40% of individuals across all age groups [8]. Its prevalence may be even greater in older HF patients, as anemia incidence rises with age. The prognostic impact of anemia in HF remains controversial. Although some studies suggest a deleterious effect on outcomes [16, 17, 18], others have not corroborated this finding [19, 20, 21]. The etiology of anemia in HF is multifactorial, involving renal impairment, iron metabolism disturbances, impaired bone marrow function, disrupted erythropoietin synthesis and response, and activation of neurohormonal and proinflammatory pathways [22, 23, 24]. One proposed key mechanism is a blunted erythropoietin response secondary to HF-induced renal impairment [23]. Anemia reduces oxygen delivery, eliciting hemodynamic and neurohormonal alterations in HF patients [25]. The consequent elevation in cardiac workload may further stimulate sympathetic nervous activity, potentially driving ventricular hypertrophy and myocardial remodeling, and ultimately increasing morbidity and mortality risk [8, 26]. Older anemic patients often coexist with comorbidities such as hypoproteinemia, impaired nutritional status, CKD, diabetes, frailty, and cardiac cachexia, all of which may contribute to adverse outcomes [9, 27]. Furthermore, anemia is often accompanied by a dysregulated proinflammatory and neurohormonal status, which can negatively influence HF prognosis [28].

Renal impairment is another frequent complication in HF patients. It has been estimated that 63% of HF patients exhibit some degree of renal dysfunction, with 29% presenting with severe CKD [29]. Evidence suggests that this prevalence may be underestimated [30]. Venous congestion and decreased renal perfusion are considered major contributors to kidney impairment in HF [31]. Additional factors, including HF progression, anemia, RAAS stimulation and increased sympathetic activity, also promote renal dysfunction [5, 32]. Converging evidence indicates that renal dysfunction is associated with increased risk of mortality and morbidity in HF patients [31, 33]. Renal dysfunction, particularly CKD, can induce various cardiac abnormalities, such as accelerated atherosclerosis, microvessel disease, coronary endothelial dysfunction, depleted cardiac energy reserves, and sympathetic overactivation [34].

HCR was first introduced by Numasawa in 2020 [35], who observed an inverse correlation between HCR and in-hospital mortality and bleeding complications among non-dialysis patients undergoing percutaneous coronary intervention (PCI). Because HCR can be easily obtained from routine laboratory tests without requiring external technology, and given the high prevalence of anemia and renal impairment across many diseases, this composite indicator has attracted considerable research interest. In a study by Demir and colleagues, a higher HCR was found to be associated with a lower 5-year mortality rate in patients with acute coronary syndrome [36]. Another study assessed the association between HCR and contrast induced nephropathy in PCI patients and found that HCR was an independent predictor for this complication [37]. Moreover, a recent study demonstrated that elevated HCR is associated with reduced one-year mortality and HF hospitalization rates after transcatheter aortic valve implantation [38]. Collectively, these studies indicate that higher HCR levels are associated with a lower risk of adverse prognosis compared to lower levels. In the present study, we also found that a higher HCR was independently associated with decreased risk of readmission or death within 180 days of discharge in elderly HF patients. This association remained robust across multiple sensitivity analyses and most subgroups. The mechanism linking HCR to outcomes in HF patients remains incompletely understood. As established previously, both anemia and renal impairment frequently coexist in HF patients and accelerate disease progression by amplifying key pathological pathways such as inflammation, oxidative stress, sympathetic nervous system and RAAS stimulation [34]. When anemia and renal dysfunction arise during the course of HF, these mechanisms can be perpetuated and enhanced through positive feedback loops, progressively worsening the disease, complicating its management, and ultimately leading to a poor prognosis [11]. Thus, it may be speculated that anemia and renal impairment act synergistically to multiply the risk of poor prognosis of HF patients. Additionally, HF itself can cause progressive renal dysfunction, which may subsequently contribute to anemia. Thus, as HF progresses, the HCR is likely to increase gradually. However, it remains uncertain whether a low HCR serves as an independent prognostic marker or simply reflects overall HF severity. Based on the aforementioned evidence, the coexistence of heart failure, anemia, and renal dysfunction appears to form a pathological triangle (cardiorenal anemia syndrome) [34]. These three conditions mutually exacerbate one another, creating a vicious cycle that ultimately leads to adverse clinical outcomes in HF patients.

Given the significant burden of HF, accurate risk stratification after hospital discharge is important for optimizing patient management. The prognostic value of HCR was further underscored by ROC analysis. Notably, HCR demonstrated a comparable discriminatory ability to the established biomarker BNP. Moreover, the combination of HCR and BNP yielded a higher area under the curve (AUC), suggesting that HCR provides complementary prognostic information that is not fully captured by BNP alone. Given that HCR is derived from routine, low-cost blood tests, it is readily available in virtually all clinical settings without additional financial burden. Thus, HCR may serve as a practical and cost-effective prognostic marker for post-discharge risk assessment in elderly HF patients, with potential utility in guiding out-hospital management and improving outcomes in this high-risk population. The results also underscore the importance of systematically monitoring anemia and renal function in HF patients to facilitate early identification and timely intervention, which could further optimize HF management and improve outcomes. Although single HCR measurements show prognostic value, longitudinal tracking may offer additional insights into disease progression. These findings position HCR as both a promising risk-stratification tool and a reminder of the value of integrated anemia-renal assessment in comprehensive HF care. Further studies are warranted to validate these potential benefits.

Subgroup analysis revealed a significant interaction effect across age groups, indicating that the association between HCR and post-discharge prognosis is not uniform across the elderly population.

This may be partly explained by the escalating prevalence of frailty and sarcopenia in the oldest-old population. Age-related reduction in muscle mass leads to a spuriously lower serum creatinine level, thereby decoupling creatinine from true renal function. In this context, the HCR may be transformed from an integrative cardio-renal biomarker into a potential surrogate marker for frailty and sarcopenic status, which are powerful independent predictors of poor outcomes. Clinicians should therefore be cautious when applying HCR for risk stratification in very old and potentially frail patients.

## 5. Limitations

Several limitations of this study deserve mention. First, the study population was recruited from a single center in Zigong, China, which may limit the generalizability of our findings. Second, despite adjustment for several covariates in regression models, residual confounding may persist due to unmeasured factors such as frailty, inflammatory markers, nutritional status, iron metabolism markers, psychosomatic state, and socioeconomic status. Moreover, data on iron supplementation, erythropoietin-stimulating agents, and nephroprotective drug use were lacking in this database, potentially influencing the observed associations. Third, as a retrospective analysis, the study can only indicate an association between HCR and post-discharge outcomes rather than establish causality. Consequently, HCR should be viewed as a risk stratification tool rather than a modifiable target. Fourth, the database does not include data on HF type, such as acute/chronic status or other types. Given the distinct characteristics of HF subtypes, the association between HCR and outcomes might be differentially modified by HF type. Further investigation is warranted to elucidate the prognostic utility of HCR across these subtypes. Fifth, the high missingness of LVEF may bias the results. Nevertheless, a sensitivity analysis restricted to patients with available LVEF data yielded results consistent with our primary findings, strengthening the robustness of the observed association between HCR and outcomes. Future studies should implement stricter protocols to minimize missing data through prospective design and standardized data collection. Finally, the underlying causes of renal impairment and anemia were not available in this database. Notwithstanding these limitations, this study contributes meaningfully to identifying appropriate indicators for distinguishing older HF patients at elevated risk of adverse outcomes after discharge. Future large-scale, multi-center studies involving more diverse patient cohorts are warranted to validate our findings. Moreover, interventional studies are needed to determine whether targeting anemia and renal dysfunction can improve outcomes in this vulnerable population.

## 6. Conclusions

The study found an inverse association between HCR and the risk of readmission or mortality within 180 days after hospital discharge in elderly HF patients. HCR may serve as a simple and cost-effective method for stratifying risk in elderly HF patients who survive to discharge.

## Availability of Data and Materials

Data from this study are available at the following link: https://physionet.org/content/heart-failure-zigong/1.2/.
