# Supplementary material for: Serum Hemoglobin-to-Creatinine Ratio and Post-Discharge Readmission or Mortality in Older Patients With Heart Failure: A Retrospective Cohort Study
Source: Rev Cardiovasc Med. 2026 May 6;27(5):45668. doi: 10.31083/RCM45668 (PMC13227392; doi:10.31083/RCM45668)

**Supplementary materials**

Supplementary Table 1. Subgroup analysis of the association of HCR and 90-day readmission or mortality after hospital discharge

| Subgroup | Events (%) | HR (95% CI) | *P* for interaction |
| --- | --- | --- | --- |
| Sex |  |  |  |
| Female | 276 (26) | 0.92 (0.75-1.13) | 0.029 |
| Male | 204 (28.3) | 0.68 (0.48-0.95) |  |
| Age |  |  | 0.007 |
| ≥ 80 | 201 (27.5) | 1.27 (0.96-1.67) |  |
| < 80 | 279 (26.5) | 0.66 (0.53-0.83) |  |
| BMI |  |  |  |
| <18.5 | 132 (28.5) | 0.88 (0.63-1.23) | 0.922 |
| 18.5-24 | 259 (27.3) | 0.85 (0.67-1.09) |  |
| ≥24 | 89 (24.2) | 0.84 (0.57-1.25) |  |
| NYHA classification |  |  |  |
| II | 58 (18.8) | 1.01 (0.62-1.65) | 0.115 |
| III | 229 (24.9) | 0.80 (0.63-1.01) |  |
| IV | 193 (34.8) | 0.97 (0.72-1.32) |  |
| LVEF |  |  | 0.518 |
| ≥50% | 84 (26.9) | 0.85 (0.53-1.36) |  |
| <50% | 61 (24.9) | 0.91 (0.51-1.62) |  |
| Missing | 335 (27.4) | 0.86 (0.70-1.05) |  |
| CCI |  |  |  |
| <2 | 180 (25.4) | 0.68 (0.52-0.90) | 0.148 |
| ≥2 | 300 (28) | 0.97 (0.78-1.22) |  |
| Diabetes |  |  |  |
| No | 347 (25.5) | 0.71 (0.58-0.88) | 0.067 |
| Yes | 133 (31.5) | 1.19 (0.89-1.61) |  |
| COPD |  |  |  |
| No | 418 (26.8) | 0.87 (0.72-1.04) | 0.738 |
| Yes | 62 (28.2) | 0.81 (0.45-1.45) |  |
| Dementia |  |  |  |
| No | 450 (27) | 0.85 (0.71-1.02) | 0.724 |
| Yes | 30 (26.8) | 0.70 (0.30-1.61) |  |
| Cerebrovascular disease |  |  |  |
| No | 441 (26.7) | 0.80 (0.67-0.96) | 0.551 |
| Yes | 39 (29.8) | 1.20 (0.61-2.38) |  |

Abbreviations: HCR, hemoglobin-creatinine ratio; HR, hazard ratio; CI, confidence interval; BMI, body max index; NYHA classification, New York heart association classification; CCI, Charlson Comorbidity Index; COPD, chronic obstructive pulmonary disease.

Supplementary Table 2*. Results of Cox regression between HCR and composite outcome in patients with heart failure

|  | Event (%) | Model 1 | |  | Model 2 | |  | Model 3 | |
| --- | --- | --- | --- | --- | --- | --- | --- | --- | --- |
|  |  | HR (95% CI) | *P* value |  | HR (95% CI) | *P* value |  | HR (95% CI) | *P* value |
| 90-day readmission or mortality after hospital discharge | | | |  |  |  |  |  |  |
| HCR | 435 (26.5) | 0.69 (0.60~0.80) | <0.001 |  | 0.69 (0.59~0.80) | <0.001 |  | 0.83 (0.69~0.99) | 0.043 |
| Tertiles of HCR |  |  |  |  |  |  |  |  |  |
| T1 | 192 (35.2) | Reference |  |  | Reference |  |  | Reference |  |
| T2 | 141 (25.6) | 0.68 (0.55~0.85) | 0.001 |  | 0.68 (0.55~0.85) | 0.001 |  | 0.75 (0.59~0.95) | 0.02 |
| T3 | 102 (18.6) | 0.48 (0.38~0.61) | <0.001 |  | 0.47 (0.37~0.61) | <0.001 |  | 0.59 (0.44~0.78) | <0.001 |
| P for trend |  |  | <0.001 |  |  | <0.001 |  |  | <0.001 |
| 180-day readmission or mortality after hospital discharge | | | |  |  |  |  |  |  |
| HCR | 677 (41.2) | 0.71 (0.63~0.8) | <0.001 |  | 0.71 (0.63~0.79) | <0.001 |  | 0.79 (0.68~0.91) | 0.001 |
| Tertiles of HCR |  |  |  |  |  |  |  |  |  |
| T1 | 281 (51.5) | Reference |  |  | Reference |  |  | Reference |  |
| T2 | 221 (40.2) | 0.69 (0.56~0.86) | 0.001 |  | 0.70 (0.59~0.84) | <0.001 |  | 0.74 (0.60~0.90) | 0.002 |
| T3 | 175 (32) | 0.56 (0.45~0.70) | <0.001 |  | 0.52 (0.43~0.63) | <0.001 |  | 0.58 (0.46~0.74) | <0.001 |
| P for trend |  |  | <0.001 |  |  | <0.001 |  |  | <0.001 |

Model 1 adjusted for none; Model 2 adjusted for age, sex, and BMI; Model 3 adjusted for age, sex, BMI, CCI, LVEF, NYHA classification, MAP, diabetes, Sodium, potassium, albumin, BNP, bilirubin, loop diuretics, RAS inhibitor, beta blocker, inotropes.

Abbreviations: HCR, hemoglobin-creatinine ratio; HR, Hazard ratio; BMI, body mass index; CI, confidence interval; CCI, Charlson Comorbidity Index; LVEF, left ventricular ejection fraction, NYHA, New York heart association; MAP, mean arterial pressure; BNP, brain natriuretic peptide; RAS, renin-angiotensin-aldosterone system.

* Heart failure patients with missing values (n=138) were excluded, and 1643 patients were included for analysis.

Supplementary Table 3*. Results of Cox regression between HCR and composite outcome in patients with heart failure

|  | Event (%) | Model 1 | |  | Model 2 | |  | Model 3 | |
| --- | --- | --- | --- | --- | --- | --- | --- | --- | --- |
|  |  | HR (95% CI) | *P* value |  | HR (95% CI) | *P* value |  | HR (95% CI) | *P* value |
| 90-day readmission or mortality after hospital discharge | | | |  |  |  |  |  |  |
| HCR | 332 (24.6) | 0.71 (0.60~0.85) | <0.001 |  | 0.71 (0.59~0.85) | <0.001 |  | 0.79 (0.65~0.95) | 0.015 |
| Tertiles of HCR |  |  |  |  |  |  |  |  |  |
| T1 | 108 (35.9) | Reference |  |  | Reference |  |  | Reference |  |
| T2 | 117 (24.2) | 0.63 (0.49~0.82) | 0.001 |  | 0.63 (0.48~0.81) | <0.001 |  | 0.66 (0.50~0.87) | 0.003 |
| T3 | 107 (19) | 0.48 (0.37~0.63) | <0.001 |  | 0.48 (0.36~0.62) | <0.001 |  | 0.54 (0.40~0.73) | <0.001 |
| P for trend |  |  | <0.001 |  |  | <0.001 |  |  | <0.001 |
| 180-day readmission or mortality after hospital discharge | | | |  |  |  |  |  |  |
| HCR | 521 (38.7) | 0.75 (0.65~0.86) | <0.001 |  | 0.74 (0.64~0.85) | <0.001 |  | 0.76 (0.65~0.89) | <0.001 |
| Tertiles of HCR |  |  |  |  |  |  |  |  |  |
| T1 | 150 (49.8) | Reference |  |  | Reference |  |  | Reference |  |
| T2 | 186 (38.5) | 0.69 (0.56~0.86) | 0.001 |  | 0.69 (0.56~0.85) | 0.001 |  | 0.69 (0.55~0.86) | 0.001 |
| T3 | 185 (32.9) | 0.56 (0.45~0.70) | <0.001 |  | 0.55 (0.44~0.69) | <0.001 |  | 0.58 (0.46~0.74) | <0.001 |
| P for trend |  |  | <0.001 |  |  | <0.001 |  |  | <0.001 |

Model 1 adjusted for none; Model 2 adjusted for age, sex, and BMI; Model 3 adjusted for age, sex, BMI, CCI, LVEF, NYHA classification, MAP, diabetes, Sodium, potassium, albumin, BNP, bilirubin, loop diuretics, RAS inhibitor, beta blocker, inotropes.

Abbreviations: HCR, hemoglobin-creatinine ratio; HR, Hazard ratio; BMI, body mass index; CI, confidence interval; CCI, Charlson Comorbidity Index; LVEF, left ventricular ejection fraction, NYHA, New York heart association; MAP, mean arterial pressure; BNP, brain natriuretic peptide; RAS, renin-angiotensin-aldosterone system.

* Heart failure patients with chronic kidney disease (n=434) were excluded, and 1347 patients were included for analysis.

Supplementary Table 4*. Results of Cox regression between HCR and composite outcome in patients with heart failure

|  | Event (%) | Model 1 | |  | Model 2 | |  | Model 3 | |
| --- | --- | --- | --- | --- | --- | --- | --- | --- | --- |
|  |  | HR (95% CI) | *P* value |  | HR (95% CI) | *P* value |  | HR (95% CI) | *P* value |
| 90-day readmission or mortality after hospital discharge | | | |  |  |  |  |  |  |
| HCR | 145 (26.0) | 0.63 (0.48~0.81) | <0.001 |  | 0.64 (0.49~0.85) | 0.002 |  | 0.79 (0.55~1.14) | 0.207 |
| Tertiles of HCR |  |  |  |  |  |  |  |  |  |
| T1 | 66 (35.9) | Reference |  |  | Reference |  |  | Reference |  |
| T2 | 44 (24.2) | 0.62 (0.42~0.9) | 0.013 |  | 0.62 (0.42~0.91) | 0.014 |  | 0.66 (0.42~1.03) | 0.070 |
| T3 | 35 (18.3) | 0.46 (0.3~0.69) | <0.001 |  | 0.49 (0.32~0.75) | 0.001 |  | 0.58 (0.34~0.97) | 0.039 |
| P for trend |  |  | <0.001 |  |  | 0.001 |  |  | 0.039 |
| 180-day readmission or mortality after hospital discharge | | | |  |  |  |  |  |  |
| HCR | 234 (42.0) | 0.66 (0.54~0.81) | <0.001 |  | 0.67 (0.54~0.83) | <0.001 |  | 0.73 (0.55~0.96) | 0.026 |
| Tertiles of HCR |  |  |  |  |  |  |  |  |  |
| T1 | 97 (52.7) | Reference |  |  | Reference |  |  | Reference |  |
| T2 | 77 (42.3) | 0.70 (0.52~0.95) | 0.02 |  | 0.70 (0.52~0.95) | 0.022 |  | 0.73 (0.51~1.05) | 0.090 |
| T3 | 60 (31.4) | 0.49 (0.35~0.67) | <0.001 |  | 0.50 (0.36~0.70) | <0.001 |  | 0.54 (0.36~0.82) | 0.004 |
| P for trend |  |  | <0.001 |  |  | <0.001 |  |  | 0.004 |

Model 1 adjusted for none; Model 2 adjusted for age, sex, and BMI; Model 3 adjusted for age, sex, BMI, CCI, LVEF, NYHA classification, MAP, diabetes, Sodium, potassium, albumin, BNP, bilirubin, loop diuretics, RAS inhibitor, beta blocker, inotropes.

Abbreviations: HCR, hemoglobin-creatinine ratio; HR, Hazard ratio; BMI, body mass index; CI, confidence interval; CCI, Charlson Comorbidity Index; LVEF, left ventricular ejection fraction, NYHA, New York heart association; MAP, mean arterial pressure; BNP, brain natriuretic peptide; RAS, renin-angiotensin-aldosterone system.

* Heart failure patients with missing LVEF were excluded (n=1224) were excluded, and 557 patients were included for analysis.

Supplementary Table 5. ROC curve analysis of HCR, BNP and the combination of HCR and BNP.

| index | AUC | 95% CI | Sensitivity (%) | Specificity (%) |
| --- | --- | --- | --- | --- |
| HCR | 0.585 | 0.559-0.612 | 46.2 | 68.3 |
| BNP | 0.544 | 0.516-0.571 | 58.2 | 52.3 |
| HCR+BNP | 0.591 | 0.565-0.618 | 55.9 | 59.5 |

Abbreviations: HCR, hemoglobin-creatinine ratio; BNP, brain natriuretic peptide; ROC, receiver operating characteristic.

Supplementary Fig. 1. ROC curves for the prediction of 180-day mortality or readmission after hospital discharge in elderly HF patients.


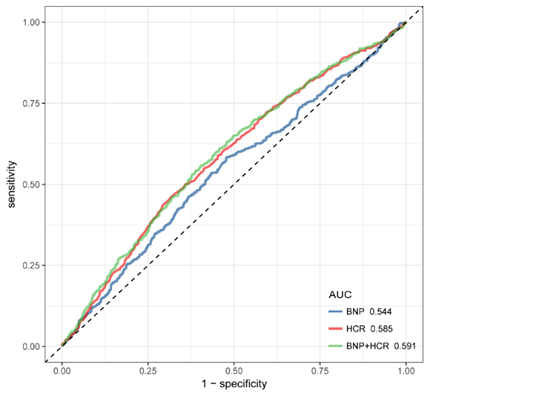

Supplement: Supplementary file 1 [file 2153-8174-27-5-45668-s1.zip › Supplementary Material.docx]
